# Public Health Measures During the COVID-19 Pandemic Reduce the Spread of Other Respiratory Infectious Diseases

**DOI:** 10.3389/fpubh.2021.771638

**Published:** 2021-11-10

**Authors:** Cheng-yi Hu, Yu-wen Tang, Qi-min Su, Yi Lei, Wen-shuai Cui, Yan-yan Zhang, Yan Zhou, Xin-yan Li, Zhong-fang Wang, Zhu-xiang Zhao

**Affiliations:** ^1^Department of Infectious Diseases, Guangzhou First People's Hospital, School of Medicine, South China University of Technology, Guangzhou, China; ^2^State Key Laboratory of Respiratory Disease & National Clinical Research Center for Respiratory Disease, Guangzhou Institute of Respiratory Health, The First Affiliated Hospital of Guangzhou Medical University, Guangzhou Medical University, Guangzhou, China

**Keywords:** COVID-19, public health measures, respiratory infectious diseases, infectious diseases, measles, tuberculosis, influenza

## Abstract

**Background:** Public health measures (such as wearing masks, physical distancing, and isolation) have significantly reduced the spread of the coronavirus disease-2019 (COVID-19), but the impact of public health measures on other respiratory infectious diseases is unclear.

**Objective:** To assess the correlation between public health measures and the incidence of respiratory infectious diseases in China during the COVID-19 pandemic.

**Methods:** We collected the data from the National Health and Construction Commission in China on the number of patients with six respiratory infectious diseases (measles, tuberculosis, pertussis, scarlet fever, influenza, and mumps) from 2017 to 2020 and assessed the correlation between public health measures and the incidence of respiratory infectious diseases. Finally, we used the data of the six respiratory infectious diseases in 2021 to verify our results.

**Results:** We found public health measures significantly reduced the incidence of measles (*p* = 0.002), tuberculosis (*p* = 0.002), pertussis (*p* = 0.004), scarlet fever (*p* = 0.002), influenza (*p* = 0.034), and mumps (*p* = 0.002) in 2020, and prevented seasonal peaks. Moreover, the effects of public health measures were most marked during the peak seasons for these infections. Of the six respiratory infectious diseases considered, tuberculosis was least affected by public health measures.

**Conclusion:** Public health measures were very effective in reducing the incidence of respiratory infectious diseases, especially when the respiratory infectious diseases would normally have been at their peak.

## Introduction

The coronavirus disease-2019 (COVID-19) pandemic has severely affected economic development worldwide. To reduce the transmission of COVID-19, various types of public health measures have been implemented worldwide, including wearing masks, physical distancing, strict closed-off management, strict immigration control measures, and isolation (1–3). These public health measures have been effective in reducing the spread of COVID-19 (4, 5) and have also reduced the spread of other infectious diseases.

A recent report claimed that public health measures reduced the activity of enveloped viruses (6). This phenomenon was validated by other independent studies from different countries (5, 7, 8). In Singapore, the number of influenza cases decreased by 76% during the fifth to sixth epidemiologic weeks, compared with that of 2016–2019 (9). In China, public health measures have minimized influenza transmission since the sixth epidemiologic week (10). However, the impact of public health measures on other respiratory pathogens has not been fully investigated.

It is useful to evaluate the impact of public health measures on respiratory infectious diseases in China. The first case of COVID-19 was reported in China by the Wuhan Municipal Health Commission at the end of 2019. Starting from January 2020, people were asked to wear masks in crowded closed places and public places, reduce participation in non-essential gatherings, and maintain a social distance of more than 1 meter. All provinces in mainland China adopted stringent public health measures from January 2020 (11). Taking Wuhan as an example, on January 14, 2020, the government implemented the entry and exit policy, and established temperature detection points in public places. Subsequently, the city was placed under lockdown on January 23 by closing national roads, waterways, and passenger flights in and out of Wuhan. The COVID-19 pandemic was controlled in April 2020 (12). Starting from April 8th, the city was not on lockdown. Until May 25, 2020, the government announced that students on school campuses in low-risk areas outside Wuhan need not wear masks, and teachers were not required to wear masks when teaching; however, students, faculty, and staff in Wuhan were required to continue to wear masks and maintain a social distance of more than 1 m. Most Chinese people have continued wearing masks to date. Considering that strict public health measures were implemented in China over a clearly defined time period, this provided the opportunity for us to conduct a retrospective study to determine the effect of public health measures on the transmission of respiratory infectious diseases.

## Materials and Methods

Taking into account the differences in pathogen types, transmission routes, epidemic cycles, and basic reproduction number (R_0_) of respiratory infectious diseases, we collected the data on the number of patients with six respiratory infectious diseases (measles, tuberculosis, pertussis, scarlet fever, influenza, and mumps) for each month from 2017 to 2021 from the National Health and Construction Commission (http://www.nhc.gov.cn/). The incidence of these respiratory infectious diseases in China was high enough that the relationship between public health measures and the transmission of respiratory infectious diseases could be well-reflected.

Considering that no public health measures, such as wearing masks, inter-city traffic controls, and restrictions of personal movement, were adopted from 2017 to 2019 in China, the average of monthly number of newly confirmed cases from 2017 to 2019 was used to estimate the monthly number of newly confirmed cases expected in 2020, had public health measures not been adopted in 2020. In order to assess the effect of public health measures on the transmission of respiratory infectious diseases during COVID-19 pandemic, the monthly average number of newly confirmed cases from 2017 to 2019 was compared to the number of cases reported in 2020. Finally, data on the monthly number of newly confirmed cases in 2021 were used to verify the results.

SPSS version 22.0 (IBM, Armonk, New York, USA) was used for the statistical analyses. The Shapiro–Wilk normality test was used to determine the normality of the data. Continuous variables were compared using the paired *t*-tests or Wilcoxon signed-rank tests, as appropriate. The Spearman rank correlation analysis was used to analyze the correlation between public health measures and the monthly number of newly confirmed cases.

## Results

### Baseline Number of Newly Confirmed Cases in China From 2017 to 2020

**Figure 1:**
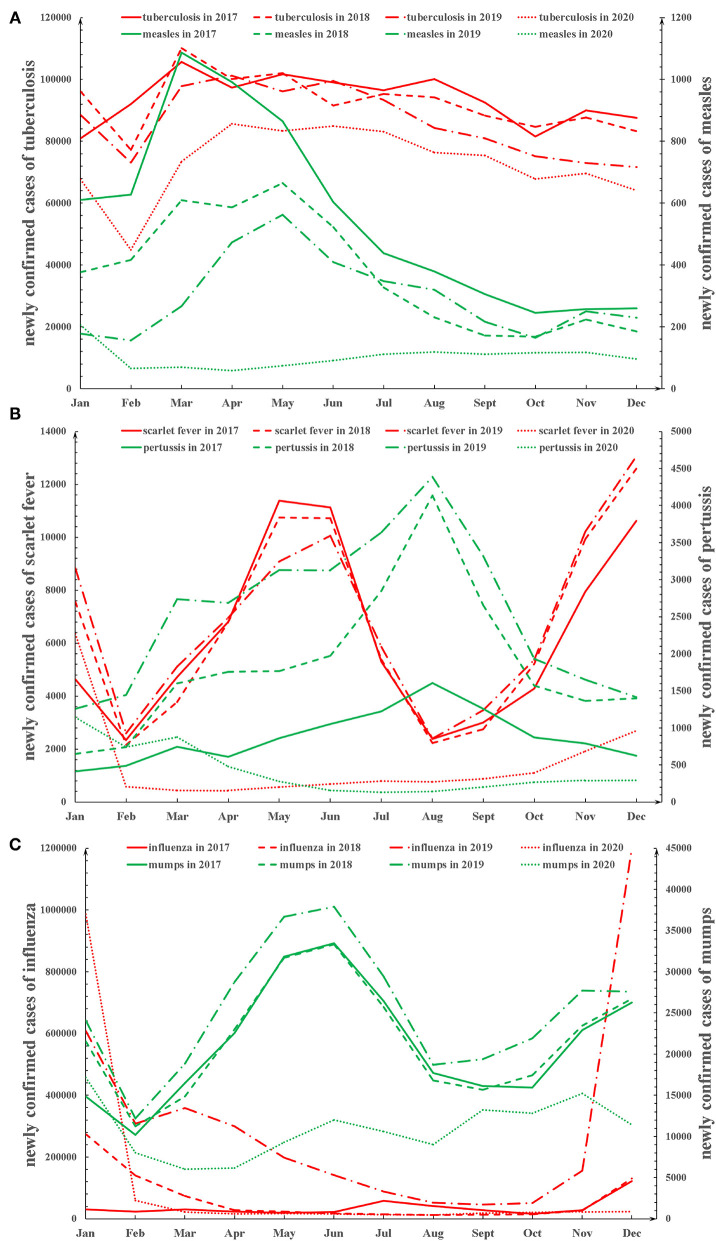
The trends of the monthly newly confirmed cases of the six respiratory infectious diseases from 2017 to 2020 in China. **(A)** The trends of the monthly newly confirmed cases of measles and tuberculosis from 2017 to 2020. **(B)** The trends of the monthly newly confirmed cases of pertussis and scarlet fever from 2017 to 2020. **(C)** The trends of the monthly newly confirmed cases of influenza and mumps from 2017 to 2020.

Figure 1 shows the trends of the monthly newly confirmed cases of measles, tuberculosis, pertussis, scarlet fever, influenza, and mumps in China from 2017 to 2020. The incidence of measles, pertussis, influenza, and mumps peaked around March, August, December, and June, respectively. No significant peak was observed in the trends of the monthly number of newly confirmed cases of tuberculosis. In addition, there were two peak periods of scarlet fever every year, in May and December. From 2017 to 2019, the peak incidence of pertussis and influenza rose each year, while the peaks in measles dropped each year. However, the peak of incidence in measles, pertussis, scarlet fever, influenza, and mumps, which were expected in 2020, disappeared due to the public health measures adopted during the COVID-19 pandemic. Supplementary Table 1 described in detail the monthly newly confirmed cases of measles, tuberculosis, pertussis, scarlet fever, influenza, and mumps in China from 2017 to 2020.

### The Effect of Public Health Measures on the Transmission of Respiratory Infectious Diseases in 2020

**Figure 2:**
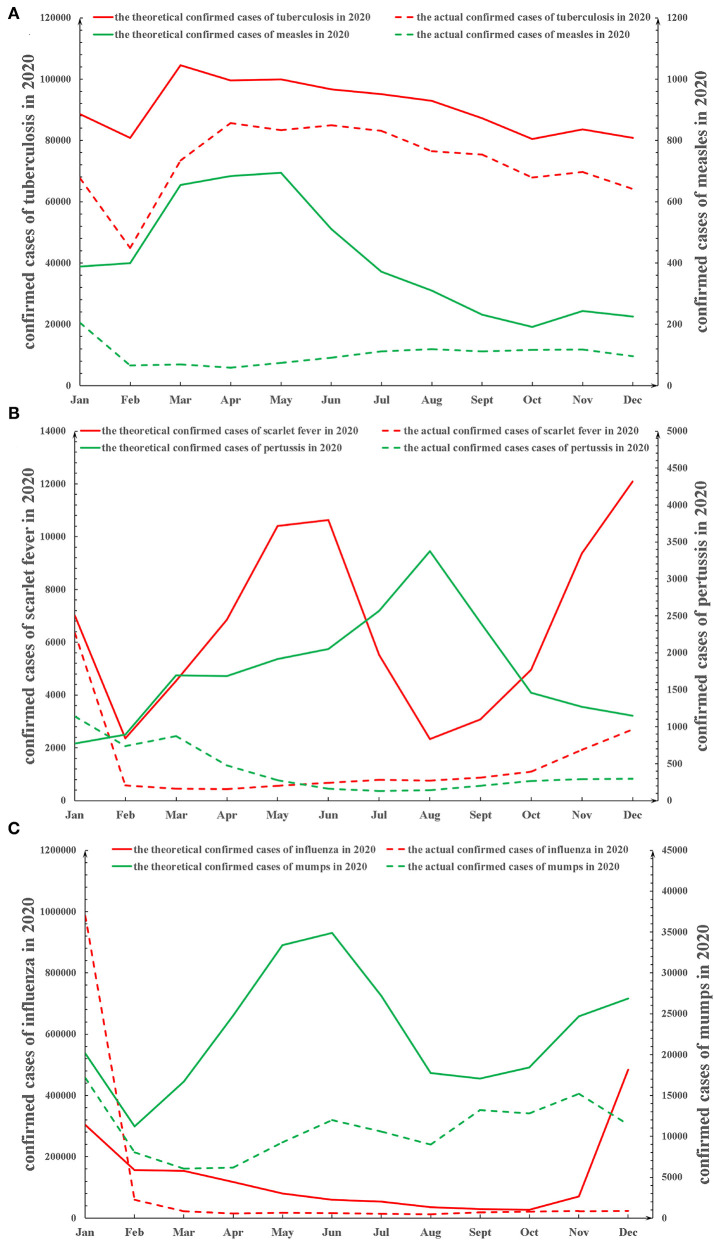
The theoretical and actual trends of monthly new confirmed cases in the six respiratory infectious diseases in 2020 in China. **(A)** The theoretical and actual trends of monthly new confirmed cases in measles and tuberculosis in 2020 in China. **(B)** The theoretical and actual trends of monthly new confirmed cases in pertussis and scarlet fever in 2020 in China. **(C)** The theoretical and actual trends of monthly new confirmed cases in influenza and mumps in 2020 in China. The average of monthly number of newly confirmed cases from 2017 to 2019 was used to estimate the theoretical monthly number of newly confirmed cases expected in 2020, had public health measures not been adopted in 2020.

As shown in Supplementary Table 2, the results of the Spearman rank correlation analysis showed that there was a significantly negative correlation between public health measures and the monthly number of newly confirmed cases of measles (*p* < 0.001), tuberculosis (*p* < 0.001), pertussis (*p* < 0.001), scarlet fever (*p* < 0.001), influenza (*p* = 0.013), and mumps (*p* < 0.001). Compared with the projected monthly number of newly confirmed cases that would have occurred in 2020 in the absence of public health measures, the actual monthly number of newly confirmed cases was significantly reduced for measles (*p* = 0.002), tuberculosis (*p* = 0.002), pertussis (*p* = 0.004), scarlet fever (*p* = 0.002), influenza (*p* = 0.034), and mumps (*p* = 0.002; Figure 2).

Moreover, public health measures were most effective against respiratory infectious diseases during the periods when they would normally be at the peak of their incidence. For example (Supplementary Figure 1), the decreased incidence rate of monthly newly confirmed cases of measles, reduced by public health measures in 2020, reached a peak of approximately 90% in March which is normally the peak period for measles. Similar results were found for pertussis and influenza. Although there are normally two peak periods for scarlet fever each year, the decrease in the number of monthly newly confirmed cases of scarlet fever in 2020 was most marked in May 2020 with a reduction in incidence of approximately 95%. The decreased monthly incidence in the number of newly confirmed cases in 2020 was less marked for tuberculosis than for the other five infectious diseases (Supplementary Figure 1): measles > tuberculosis (*p* = 0.002), pertussis > tuberculosis (*p* = 0.023), scarlet fever > tuberculosis (*p* = 0.003), influenza > tuberculosis (*p* = 0.034), and mumps > tuberculosis (*p* = 0.009).

Furthermore, the actual incidence of measles, tuberculosis, pertussis, scarlet fever, influenza, and mumps throughout the year in 2020 decreased by 74.8, 19.6, 76.5, 78.3, 22.0, and 52.1%, respectively (Supplementary Table 3). The impact of public health measures on respiratory infectious diseases was possibly related to a decrease in the R_0_ of each disease.

### Verifying the Effect of Public Health Measures on the Transmission of Respiratory Infectious Diseases in 2021

To verify our results, we collected the data from the National Health and Construction Commission in China on the number of patients with the six respiratory infectious diseases from January to July 2021. In terms of the monthly number of newly confirmed cases from January to July (Supplementary Figure 2), there was no significant difference between 2021 and 2020 of measles (*p* = 0.176), tuberculosis (*p* = 0.499), pertussis (*p* = 0.482), scarlet fever (*p* = 0.237), influenza (*p* > 0.999), or mumps (*p* = 0.819). The monthly number of newly confirmed cases of the six respiratory infectious diseases remained at a low level in 2021. No significant epidemic peaks have been observed in the six respiratory infectious diseases in 2021, to date. Therefore, we speculate that the impact of public health measures during the COVID-19 pandemic on these six respiratory infectious diseases are likely to be maintained until at least the end of 2021, even though many public health measures were gradually eased from April 2020.

## Discussion and Conclusions

Our results show that public health measures significantly reduced the incidence of measles, tuberculosis, pertussis, scarlet fever, influenza, and mumps. Due to the public health measures, the seasonal peaks in the incidence of the six respiratory infectious diseases disappeared in 2020 and 2021. Public health measures were most effective against respiratory infectious diseases during the months when seasonal incidence normally peaked. Overall, our research provides new evidence for the prevention and control of respiratory infectious diseases. Considering that the COVID-19 pandemic has not yet ended, the impact of public health measures on respiratory infectious diseases still needs further attention.

It is worth noting that although public health measures were the main reason for the reduction in the incidence of respiratory infectious diseases, there were still some other confounding factors. First, people actively avoided going to hospitals during the COVID-19 pandemic because they were worried about contracting COVID-19 (13), which could cause the number of cases of respiratory infectious diseases diagnosed to be lower than the actual incidence. Second, the closed-off management during the COVID-19 pandemic would make it inconvenient for some patients to go to the hospital for treatment, resulting in a decline in the number of confirmed patients. In summary, the number of outpatient visits in some hospitals declined in China during the COVID-19 pandemic. However, as the COVID-19 pandemic was controlled in April 2020 in China, clinical diagnosis and treatment since then had basically returned to normal. Judging from the results in our study after April 2020, the incidence of the six respiratory infectious diseases was still significantly reduced, which further proved the reliability of our results. Third, some patients might have received drugs for some conditions that had a certain degree of antiviral activity, such as eye drops and ophthalmic ointments (14), which would reduce the incidence of viral respiratory disease. However, pertussis, scarlet fever and tuberculosis were not viruses while their incidence was still reduced, which proved that the use of these drugs might not be the main reason for the reduction in the incidence of these respiratory infectious diseases. Fourth, there might also be a very small number of patients who died from COVID-19 before being diagnosed with other respiratory infections.

Among the six respiratory infectious diseases that we considered, the public health measures had the least impact on tuberculosis. This can be attributed to the following factors: First, as tuberculosis has a long latency period and its diagnosis requires time, diagnosis is often delayed in China due to the technical difficulties in detecting tuberculosis. Therefore, the impact of public health measures on tuberculosis may have been delayed in the reporting system. Second, in addition to transmission through the respiratory tract, tuberculosis may also be transmitted through other routes (15–18), such as gastrointestinal and contact. However, public health measures during the COVID-19 pandemic, such as wearing masks and isolation, were mainly able to block the respiratory tract transmission route, and had little impact on other transmission routes.

Public health measures during the COVID-19 pandemic have caused tremendous changes in the incidence of other diseases. In addition to reduce respiratory infectious diseases, public health measures were related to the increase in the incidence of “quarantine dry eye” (19). In addition, studies found that the incidence of gastrointestinal viral infections was significantly reduced during the COVID-19 pandemic, such as norovirus and group A rotavirus (20, 21).

Our study had several limitations. First, we could not determine which public health measure might be the most effective in curbing the spread of respiratory infectious diseases. Second, because public health measures may change according to the situation, the degree of impact of public health measures on respiratory infectious diseases would also change accordingly. Third, we could only obtain data from China, and data from more countries need to be included to improve the reliability of the conclusions.

In conclusion, public health measures can effectively reduce the spread of respiratory infectious diseases. During an epidemic of these respiratory infectious diseases, it would be necessary to take appropriate public health measures to reduce the incidence. The findings of this study could provide evidence for tailoring control strategies for future epidemics or pandemics of respiratory infectious diseases.

## Data Availability Statement

The original contributions presented in the study are included in the article/Supplementary Material, further inquiries can be directed to the corresponding author/s.

## Author Contributions

CH and ZZ designed the research study, collected and analyzed the data, and wrote and revised the paper. YZho, XL, QS, ZW, and YT analyzed the data and revised the manuscript. YL, WC, and YZha collected the data and revised the manuscript. All authors contributed to the article and approved the submitted version.

## Funding

This work was supported by the Natural Science Foundation of Guangdong Province (Grant No. 2017A030313860).

## Conflict of Interest

The authors declare that the research was conducted in the absence of any commercial or financial relationships that could be construed as a potential conflict of interest.

## Publisher's Note

All claims expressed in this article are solely those of the authors and do not necessarily represent those of their affiliated organizations, or those of the publisher, the editors and the reviewers. Any product that may be evaluated in this article, or claim that may be made by its manufacturer, is not guaranteed or endorsed by the publisher.

## Supplementary Material

Supplementary Figure 1 The decreased incidence rate of the six respiratory infectious diseases owing to public health measure in 2020.

Supplementary Figure 2 The trends of the monthly newly confirmed cases of the six respiratory infectious diseases from January to July 2021 in China. **(A)** The trends of the monthly newly confirmed cases of measles and tuberculosis from January to July 2021. **(B)** The trends of the monthly newly confirmed cases of pertussis and scarlet fever from January to July 2021. **(C)** The trends of the monthly newly confirmed cases of influenza and mumps from January to July 2021.

Supplementary Table 1 The monthly newly confirmed cases of measles, tuberculosis, pertussis, scarlet fever, influenza, and mumps in China from 2017 to 2020.

Supplementary Table 2 The results of the Spearman rank correlation analysis between public health measures and the monthly newly confirmed cases in measles, tuberculosis, pertussis, scarlet fever, influenza, and mumps.

Supplementary Table 3 Characteristics of infectious diseases.
