# Supplementary material for: Public Health Measures During the COVID-19 Pandemic Reduce the Spread of Other Respiratory Infectious Diseases
Source: Front Public Health. 2021 Nov 10;9:771638. doi: 10.3389/fpubh.2021.771638 (PMC8631357; doi:10.3389/fpubh.2021.771638)
Supplement: Supplementary Figure 1 — The decreased incidence rate of the six respiratory infectious diseases owing to public health measure in 2020. [file Data_Sheet_1.ZIP › Supplementary-Table-1.docx]

**Supplementary Table 1.** the monthly newly confirmed cases of measles, tuberculosis, pertussis, scarlet fever, influenza, and mumps in China from 2017 to 2020

| **month** | **measles** | | | | **tuberculosis** | | | | **pertussis** | | | | **scarlet fever** | | | | **influenza** | | | | **mumps** | | | |
| --- | --- | --- | --- | --- | --- | --- | --- | --- | --- | --- | --- | --- | --- | --- | --- | --- | --- | --- | --- | --- | --- | --- | --- | --- |
|  | **2017** | **2018** | **2019** | **2020** | **2017** | **2018** | **2019** | **2020** | **2017** | **2018** | **2019** | **2020** | **2017** | **2018** | **2019** | **2020** | **2017** | **2018** | **2019** | **2020** | **2017** | **2018** | **2019** | **2020** |
| **January** | 611 | 377 | 178 | 205 | 80911 | 96125 | 88597 | 67682 | 413 | 649 | 1260 | 1141 | 4633 | 7564 | 8838 | 6352 | 30109 | 273949 | 608511 | 986543 | 14865 | 21540 | 24075 | 17159 |
| **February** | 628 | 417 | 156 | 66 | 92037 | 77224 | 73096 | 44933 | 488 | 743 | 1444 | 738 | 2335 | 2159 | 2591 | 580 | 22998 | 139738 | 307892 | 59154 | 10208 | 11238 | 12169 | 8018 |
| **March** | 1087 | 609 | 267 | 69 | 105633 | 110124 | 97866 | 73427 | 748 | 1602 | 2735 | 874 | 4718 | 3774 | 5120 | 444 | 30519 | 74086 | 358757 | 21696 | 16469 | 14858 | 18827 | 6028 |
| **April** | 992 | 587 | 473 | 58 | 97296 | 100054 | 101191 | 85684 | 608 | 1758 | 2689 | 479 | 6804 | 6784 | 6964 | 442 | 23260 | 28592 | 299939 | 15396 | 22554 | 23015 | 28735 | 6169 |
| **May** | 865 | 665 | 562 | 74 | 101628 | 102063 | 96106 | 83385 | 861 | 1764 | 3129 | 277 | 11388 | 10747 | 9087 | 562 | 19085 | 22980 | 197729 | 16974 | 31840 | 31707 | 36669 | 9273 |
| **June** | 603 | 522 | 409 | 91 | 99001 | 91603 | 99555 | 84952 | 1051 | 1973 | 3128 | 159 | 11129 | 10716 | 10052 | 677 | 22313 | 16635 | 141202 | 15640 | 33458 | 33314 | 37913 | 11995 |
| **July** | 438 | 327 | 348 | 111 | 96471 | 95338 | 93318 | 83101 | 1224 | 2850 | 3635 | 131 | 5298 | 5385 | 5864 | 789 | 57694 | 14544 | 88911 | 13406 | 26431 | 25731 | 29427 | 10583 |
| **August** | 379 | 231 | 320 | 119 | 100076 | 94232 | 84304 | 76423 | 1605 | 4134 | 4388 | 142 | 2379 | 2235 | 2396 | 763 | 41625 | 12325 | 51676 | 12833 | 17691 | 16801 | 18690 | 9008 |
| **September** | 305 | 172 | 216 | 111 | 92494 | 88302 | 80973 | 75409 | 1257 | 2657 | 3319 | 201 | 3006 | 2745 | 3473 | 877 | 27467 | 13188 | 46048 | 18432 | 16142 | 15657 | 19377 | 13212 |
| **October** | 245 | 168 | 164 | 116 | 81554 | 84680 | 75123 | 67843 | 869 | 1572 | 1933 | 267 | 4279 | 5216 | 5375 | 1102 | 16049 | 14852 | 50665 | 20401 | 15936 | 17443 | 21938 | 12818 |
| **November** | 257 | 223 | 250 | 118 | 89976 | 87709 | 73000 | 69640 | 791 | 1365 | 1653 | 291 | 7947 | 9927 | 10215 | 1925 | 27731 | 26960 | 156205 | 22783 | 22922 | 23472 | 27704 | 15211 |
| **December** | 260 | 185 | 230 | 96 | 87630 | 83205 | 71631 | 64097 | 627 | 1399 | 1414 | 294 | 10622 | 12593 | 13053 | 2693 | 121800 | 130442 | 1199771 | 23546 | 26280 | 26717 | 27581 | 11437 |
