# Supplementary material for: Public Health Measures During the COVID-19 Pandemic Reduce the Spread of Other Respiratory Infectious Diseases
Source: Front Public Health. 2021 Nov 10;9:771638. doi: 10.3389/fpubh.2021.771638 (PMC8631357; doi:10.3389/fpubh.2021.771638)
Supplement: Supplementary Figure 1 — The decreased incidence rate of the six respiratory infectious diseases owing to public health measure in 2020. [file Data_Sheet_1.ZIP › Supplementary-Table-2.docx]

**Supplementary Table 2.** the results of the Spearman rank correlation analysis between public health measures and the monthly newly confirmed cases in measles, tuberculosis, pertussis, scarlet fever, influenza, and mumps

| Spearman rank correlation | r | *p* |
| --- | --- | --- |
| measles | -0.729 | <0.001 |
| tuberculosis | -0.577 | <0.001 |
| pertussis | -0.656 | <0.001 |
| scarlet fever | -0.663 | <0.001 |
| influenza | -0.358 | 0.013 |
| mumps | -0.660 | <0.001 |

r: Spearman rank correlation coefficient
