# Supplementary material for: Public Health Measures During the COVID-19 Pandemic Reduce the Spread of Other Respiratory Infectious Diseases
Source: Front Public Health. 2021 Nov 10;9:771638. doi: 10.3389/fpubh.2021.771638 (PMC8631357; doi:10.3389/fpubh.2021.771638)
Supplement: Supplementary Figure 1 — The decreased incidence rate of the six respiratory infectious diseases owing to public health measure in 2020. [file Data_Sheet_1.ZIP › Supplementary-Table-3.docx]

**Supplementary Table 3**. Characteristics of infectious diseases

| Infectious disease | Types | Transmission | Epidemic cycles | R0 | The decreased incidence rate in 2020 |
| --- | --- | --- | --- | --- | --- |
| measles | RNA virus | droplet | 1 | 12-18 | 74.8% |
| pertussis | bacillus | droplet | 1 | 12-17 | 76.5% |
| scarlet fever | coccus | droplet | 2 | / | 78.3% |
| mumps | RNA virus | droplet or contact | 2 | 4-7 | 52.1% |
| influenza | RNA virus | droplet | 1 or 2 | 1.7-2 | 22.0% |
| tuberculosis | coccus | droplet | / | 1.1892 | 19.6% |

R0: Basic reproduction number
