# Supplementary figures and images for: Public Health Measures During the COVID-19 Pandemic Reduce the Spread of Other Respiratory Infectious Diseases
Source: Front Public Health. 2021 Nov 10;9:771638. doi: 10.3389/fpubh.2021.771638 (PMC8631357; doi:10.3389/fpubh.2021.771638)

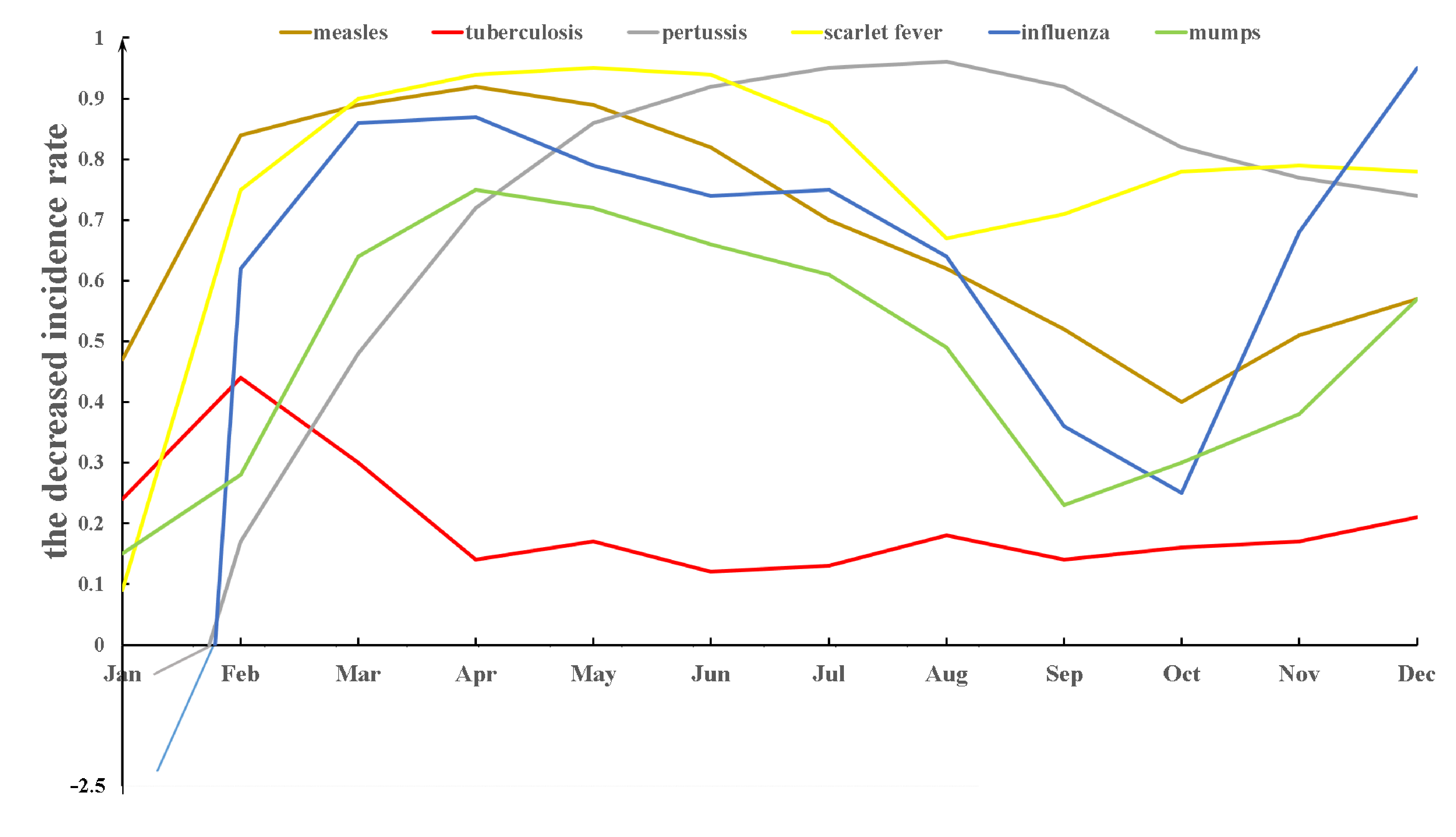

Supplement: Supplementary Figure 1 — The decreased incidence rate of the six respiratory infectious diseases owing to public health measure in 2020. [file Data_Sheet_1.ZIP › SupplementaryFigure1.tif]

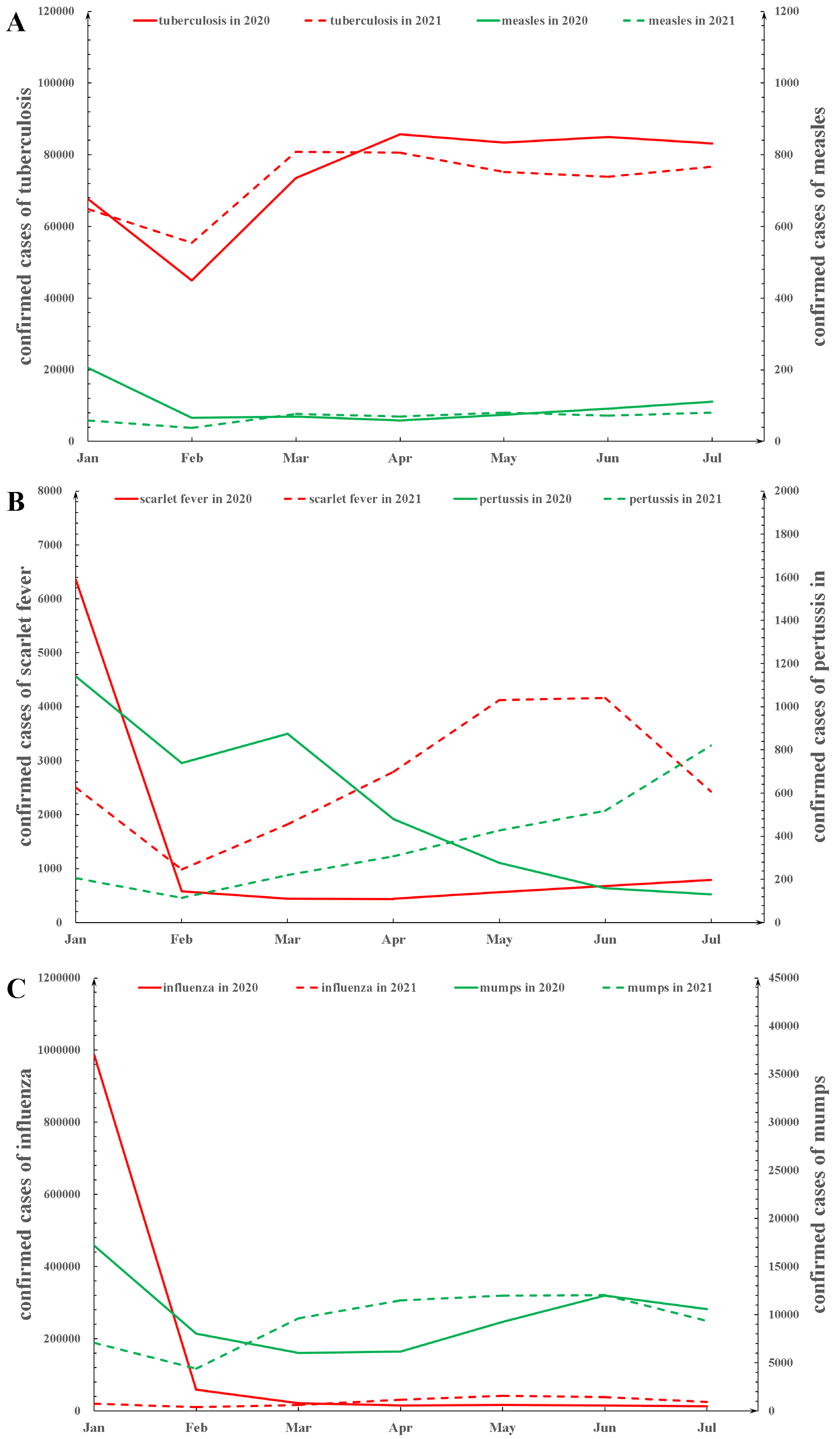

Supplement: Supplementary Figure 1 — The decreased incidence rate of the six respiratory infectious diseases owing to public health measure in 2020. [file Data_Sheet_1.ZIP › SupplementaryFigure2.tif]
